# Natural *Besnoitia besnoiti* infections in cattle: hematological alterations and changes in serum chemistry and enzyme activities

**DOI:** 10.1186/s12917-015-0326-8

**Published:** 2015-02-13

**Authors:** Martin C Langenmayer, Julia C Scharr, Carola Sauter-Louis, Gereon Schares, Nicole S Gollnick

**Affiliations:** Institute of Veterinary Pathology at the Centre for Clinical Veterinary Medicine, Ludwig-Maximilians-Universitaet Muenchen, Munich, Germany; Rammingen, Germany; Clinic for Ruminants with Ambulatory and Herd Health Services at the Centre for Clinical Veterinary Medicine, Ludwig-Maximilians-Universitaet Muenchen, Oberschleissheim, Germany; Friedrich-Loeffler-Institut, Federal Research Institute for Animal Health, Institute of Epidemiology, Greifswald-Isle of Riems, Germany

**Keywords:** Bovine besnoitiosis, Besnoitia besnoiti, Hematological and biochemical parameters, Cattle

## Abstract

**Background:**

The emerging disease bovine besnoitiosis is caused by the apicomplexan parasite *Besnoitia besnoiti*. Clinical signs of acute besnoitiosis are pyrexia, anorexia and subcutaneous edema. In subacute and chronic besnoitiosis parasitic cysts arise in a variety of tissues and affected cattle display skin lesions and weight loss. In all stages of bovine besnoitiosis, lesions can be found in many organ systems and therefore presumably alter a variety of laboratory parameters. In this study, the impact of naturally acquired acute, subacute and chronic bovine besnoitiosis on hematologic parameters, serum chemistry, and enzyme activities was investigated. Laboratory parameters of two Simmental heifers and two Limousin cows were monitored during acute, subacute and chronic besnoitiosis and in another Simmental heifer during subclinical besnoitiosis. To determine aberrations of laboratory parameters, values were compared with reference ranges obtained from *B. besnoiti* negative Simmentals (224 samples of nine animals) and Limousins (41 animals). Further, laboratory parameters of *B. besnoiti* seropositive Limousin cows (54 animals; 32 of these showing clinical signs) and healthy *B. besnoiti* seronegative Limousin cows (41 animals) were compared.

**Results:**

During acute and subacute besnoitiosis, a reduction of leukocyte and erythrocyte concentrations, hematocrit, serum albumin, urea, magnesium, and calcium concentrations were observed. Serum total protein, globulin, total bilirubin and creatinine concentrations were increased and aspartate transaminase (AST) and creatine kinase (CK) activities were elevated. In chronic besnoitiosis, erythrocyte parameters were statistically significantly lower, and total protein and globulin concentrations were significantly higher in *B. besnoiti* seropositive compared with *B. besnoiti* seronegative Limousin cows.

**Conclusions:**

In this study, altered laboratory parameters during the course of naturally acquired acute, subacute and chronic bovine besnoitiosis are described for the first time. Only a few animals were examined in acute and subacute besnoitiosis, however the alterations of laboratory parameters during these stages reflected i) the acute inflammatory state (*e.g.* high levels of serum globulin fractions), ii) clinical findings such as disturbed condition (*e.g.* bilirubin concentrations), and iii) lesions such as muscle necroses described in the literature (*e.g.* AST or CK activities). Chronic besnoitiosis led to typical alterations of chronic inflammatory diseases like hyper-(gamma)-globulinemia or reduced erythrocyte concentrations.

## Background

*Besnoitia besnoiti*, a cyst-forming apicomplexan protozoon, is the causative agent of bovine besnoitiosis. Bovine besnoitiosis has spread within Europe in the past few years, with latest outbreaks in Hungary [1], Switzerland [2], Italy [3], and Germany [4]. In 2010, the disease has been classified as “emerging disease” by the European Food Safety Authority [5]. Clinical signs of infected cattle in the early stage of the disease include fever, edema, enlarged lymph nodes, anorexia, weight loss and, in bulls, swollen and painful testes [6-8]. In the chronic stage, parasitic cysts arise in various organs, including skin, vascular walls, scleral *conjunctivae*, and other non-intestinal mucous membranes [9-11].

Clinical signs, morphological changes, pathological lesions, parasitological examinations and diagnosis of bovine besnoitiosis have been in the focus of researchers [6,9,12-16]. However, the effects of the parasitic infection on laboratory parameters during acute and chronic stages of the disease were not investigated.

*B. caprae*, a parasite very closely related to *B. besnoiti* [17,18], causes similar clinical signs in goats [19,20] and data on altered laboratory parameters in caprine besnoitiosis are already available. In naturally acquired caprine besnoitiosis in Iran, alterations of various hematological and biochemical parameters as well as enzyme activities have been observed [20,21]. However, similar examinations regarding bovine besnoitiosis are missing, and metabolic disturbances caused by tachyzoites and bradyzoites are unknown.

We hypothesized that pathological alterations during the acute stage like hemorrhages, necroses or degenerative lesions in different organs, for example muscle and liver cells, and activation of lymphatic tissues [15] lead to alterations of laboratory parameters connected to those lesions. Hemorrhages may affect erythrocyte parameters, muscle and liver necroses may lead to an increase in organ specific enzyme activities, and alterations of lymphatic tissues or multifocal inflammation may affect leukocyte parameters.

In the chronic stage, we hypothesized that emaciation and the huge number of tissue cysts partially surrounded by chronic granulomatous inflammation in different organs [4,9,22] may negatively affect serum albumin concentrations (emaciation) or elevate serum globulin concentrations (chronic inflammation).

The objective of the present study was to monitor laboratory parameters during acute, subacute and chronic naturally acquired bovine besnoitiosis and to connect those findings to already obtained clinical and serological findings in the same animals [13,23,24]. In addition, we aimed to compare these findings with laboratory parameters of *B. besnoiti* seronegative cattle and altered parameters of a larger group of chronically infected seropositive Limousin cattle from a German cow-calf-operation (Herd-BbGER1) [13,23].

## Methods

### Animals

#### Chronology of acute, subacute, and chronic bovine besnoitiosis monitoring infected Simmental and Limousin cattle

Animals were part of a longitudinal study focusing on the different stages and disease progression of naturally acquired bovine besnoitiosis [13,23]. Permission for this study was granted by the responsible authorities (Animal ethics committee, regional government of Upper Bavaria, TV Az. 55.2-54-2531-83-09). The study consisted of a 12-week cohabitation period (August 18, 2009, until November 9, 2009). Five healthy German Simmental heifers (Study Animal [SA] 3, SA 4, SA 6, SA 8, and SA 9) and one healthy German Simmental bull (SA 1) were kept on a pasture with three chronically infected and *B. besnoiti* seropositive Limousin cows. Six healthy Simmental heifers (SA 2, SA 5, SA 7, SA 10, SA 11, and SA 12) between 11 and 20 months of age were kept on a paddock 20 m away as a control group [23]. Prior to the trial, the Simmentals tested negative for *B. besnoiti* and *Neospora caninum* antibodies in immunoblots and IFAT [24].

On trial day (td) 3 and td 51, two Limousin cows (SA 20 and SA 22) of Herd-BbGER1 suspected to be in the acute stage of bovine besnoitiosis, were added to the pasture. Infection of SA 4, SA 6, SA 20, and SA 22 was confirmed clinically, histologically, and serologically. In the case of SA 8, infection was confirmed serologically (Table 1) [23]. Although SA 22 had already seroconverted on the day of admission, this day was regarded as day of seroconversion to make inter-animal comparison possible for data analyses.

**Table 1:** **Overview of** ***B*** **.** ***besnoiti*** **affected Simmental (SA 4, SA 6, and SA 8) and Limousin (SA 20 and SA 22) cattle**

**Animal ID**	**SA 4**	**SA 6**	**SA 8**	**SA 20**	**SA 22**
**Breed**	**S**	**S**	**S**	**L**	**L**
Age (months)	20	19	13	53	49
Pregnancy status	np	np	np	p	p
Study entry (td)	1	1	1	3	51
Acute stage: start (td)	36	29	-	unknown	unknown
Acute stage: end (td)	47	41	-	7	51
Seroconversion (td)	45	35	73	4	-
Chronic stage: start (td)	64	67	-	27	63
Course of disease	mild	mild	subclinical	severe	severe

SA = Study animal, S = Simmental, L = Limousin, np = non pregnant, p = pregnant, td = trial day.

The acute stage was classified according to clinical criteria. Animals had to show fever (body temperature > 39.0°C) or at least two of the following clinical signs/diagnoses of acute besnoitiosis: depression, conjunctivitis, subcutaneous edema, lymphadenitis, lameness. As soon as cysts were clinically observed in the scleral *conjunctivae*, the term ‘chronic stage’ was used (Table 1). The subacute stage was defined as stage between the end of the acute and the beginning of the chronic stage.

Blood samples were collected twice a week during the whole trial period and daily for 21 days after animals showed signs of acute besnoitiosis. On day 225, SA 4, SA 6, SA 8, SA 20, and SA 22 were bled during a routine herd health status examination.

In total, 224 samples collected from the control heifers and SA 1, SA 3, and SA 9 were used to determine reference ranges for Simmental hematology, serum chemistry and enzymes activities. Values outside of three times interquartile range from the 25th and 75th percentile were defined as outliers and eliminated. Thereafter, the reference ranges were determined non-parametrically as interval between the 2.5th and 97.5th percentile.

Hematological and biochemical parameters of SA 4, SA 6, and SA 8 were compared with these reference ranges. Values of SA 20 and SA 22 were compared with those of *B. besnoiti* seronegative Limousin cattle (see below).

#### Herd-BbGER1: monitoring of *B. besnoiti* seropositive Limousin cattle

In total, 75 samples were collected from 54 female Limousin cattle (one to eleven years old) during routine herd health status examinations in the years 2008, 2011, and 2012. The sampling of these animals was conducted according to international guidelines and national law concerning animal welfare. Limousin cattle were free of BHV-1 and BVD infection and did not show clinical signs of gastrointestinal helminthoses or lung worm disease. All animals were seropositive for *B. besnoiti* antibodies in immunoblots and IFAT [24] and 32 animals displayed cysts in the scleral *conjunctivae* and/or vaginal *vestibula*. Only single animals showed large numbers of parasitic cysts in the vaginal *vestibula* and the scleral *conjunctivae* as well as visible and palpable lesions in the skin of the trunk, limbs and udder.

Values outside of three times interquartile range from the 25th and 75th percentile were defined as outliers and eliminated. After that, the 2.5th and 97.5th percentiles were determined non-parametrically.

#### Herd-BbGER1: monitoring of *B. besnoiti* negative Limousin cattle

Samples were collected from two groups of female adult cattle of Herd-BbGER1. Fifteen samples from 14 healthy female Limousin cattle (one to eight years old) were collected during routine herd health status examinations in 2008, 2011 and 2012. The sampling of these animals was conducted according to international guidelines and national law concerning animal welfare.

Twenty-seven Limousin cows (two to 14 years old) without a history of bovine besnoitiosis were introduced into Herd-BbGER1 in spring 2013. Samples from these animals were collected as part of the quarantine health check. Limousin cattle from both groups were free of BHV-1 and BVD infection and did not show clinical signs of gastrointestinal helminthoses or lung worm disease and sera were negative for *B. besnoiti* antibodies in immunoblots and IFAT as previously described [24].

To determine Limousin reference ranges, values outside of three times interquartile range from the 25th and 75th percentile were defined as outliers and eliminated. After that, reference ranges were determined non-parametrically as interval between the 2.5th and 97.5th percentiles.

### Blood sample collection and processing

Blood samples were collected from the tail or jugular veins. Immediately after sampling, a blood smear was performed on a glass slide for differentiation count of cells. All blood samples were stored at approximately 8°C for up to 6 hours until transferred to the laboratory. Sera for antibody detection and serum protein electrophoresis were frozen at −80°C until transferred to the Friedrich-Loeffler-Institut (Wusterhausen, Germany) and Vet Med Labor GmbH (Division of IDEXX Laboratories, Ludwigsburg, Germany) for further examination. Samples for serum chemistry, hematology and enzyme activities were kept overnight at 8°C and were processed the next day.

### Biochemical analysis and hematological examination

Urea, creatinine, total protein (TP), albumin, total bilirubin, conjugated bilirubin, calcium, magnesium, phosphor, sodium, chloride and potassium concentrations, aspartate transaminase (AST), glutamate dehydrogenase (GLDH), gamma-glutamyl transpeptidase (GGT), creatine kinase (CK) activities were analyzed in sera with a Hitachi 912 Chemistry Analyzer (Boehringer Mannheim, Mannheim, Germany) in the laboratory of the Clinic for Ruminants. Globulin, albumin/globulin (A/G) ratio and unconjugated bilirubin were determined arithmetically.

A complete blood cell count was performed using EDTA-anticoagulated blood, analyzed with a hematological analyzer (pocH-100iV DIFF, Sysmex, Norderstedt, Germany). Air-dried slides for differential blood count were stained using a Pappenheim stain (Haema Schnellfaerbung, LT-SYS® Labor + Technik Eberhard Lehmann GmbH, Berlin, Germany). Mean corpuscular hemoglobin concentration (MCHC), mean corpuscular volume (MCV) and mean corpuscular hemoglobin (MCH) were determined arithmetically.

### Serum protein gel electrophoresis

Sera from SA 4, SA 6, and SA 20 were examined on the day of seroconversion, 2 days post seroconversion (*dps*), 5 *dps* and on day 225. Sera from SA 8 and SA 22 were examined likewise with the exception of serum from 2 *dps*. In addition, three sera from SA 4 and two sera from SA 6 were analyzed during the febrile phase before seroconversion. Serum protein gel electrophoresis was performed at Vet Med Labor GmbH (Division of IDEXX Laboratories, Ludwigsburg, Germany) with a semi-automated agarose gel electrophoresis system (HYDRASIS®2, Sebia, Norcross, USA).

Reference ranges were calculated from the 24 samples of SA 1 to SA 12 from the beginning and the mid of the trial as described above. In order to exclude possible effects of parasite circulation on the determined parameters in SA 4, SA 6, and SA 8, only samples taken before the calculated incubation period of 13 days [6] were used.

### Serological examinations

To detect *B. besnoiti* antibodies in serum, three tests were performed as previously described: one IFAT and two immunoblots either based on *B. besnoiti* tachyzoite or bradyzoite antigen. A reciprocal positive cut-off titer of 200 was used in IFAT and recognition of at least four of ten bands in both immunoblots was regarded specific [24]. Animals were regarded as positive if two of the three serological tests yielded a positive result.

### Statistical analysis

Hematological results, serum chemistry and enzyme activities of *B. besnoiti* seronegative and *B. besnoiti* seropositive Limousin cattle were compared statistically. Additionally, the same parameters of *B. besnoiti* seropositive subclinically affected and *B. besnoiti* seropositive clinically affected animals were compared statistically. To assess normality, D’Agostino and Pearson omnibus normality test was applied. To compare the two groups, the non-parametric Mann–Whitney-Test was performed. *P* values below 0.01 where considered significant. Data were analyzed using the software Graphpad Prism 5.04 for Windows.

## Results

### Chronology of hematologic alterations during acute, subacute, and chronic bovine besnoitiosis

**Figure 1:**
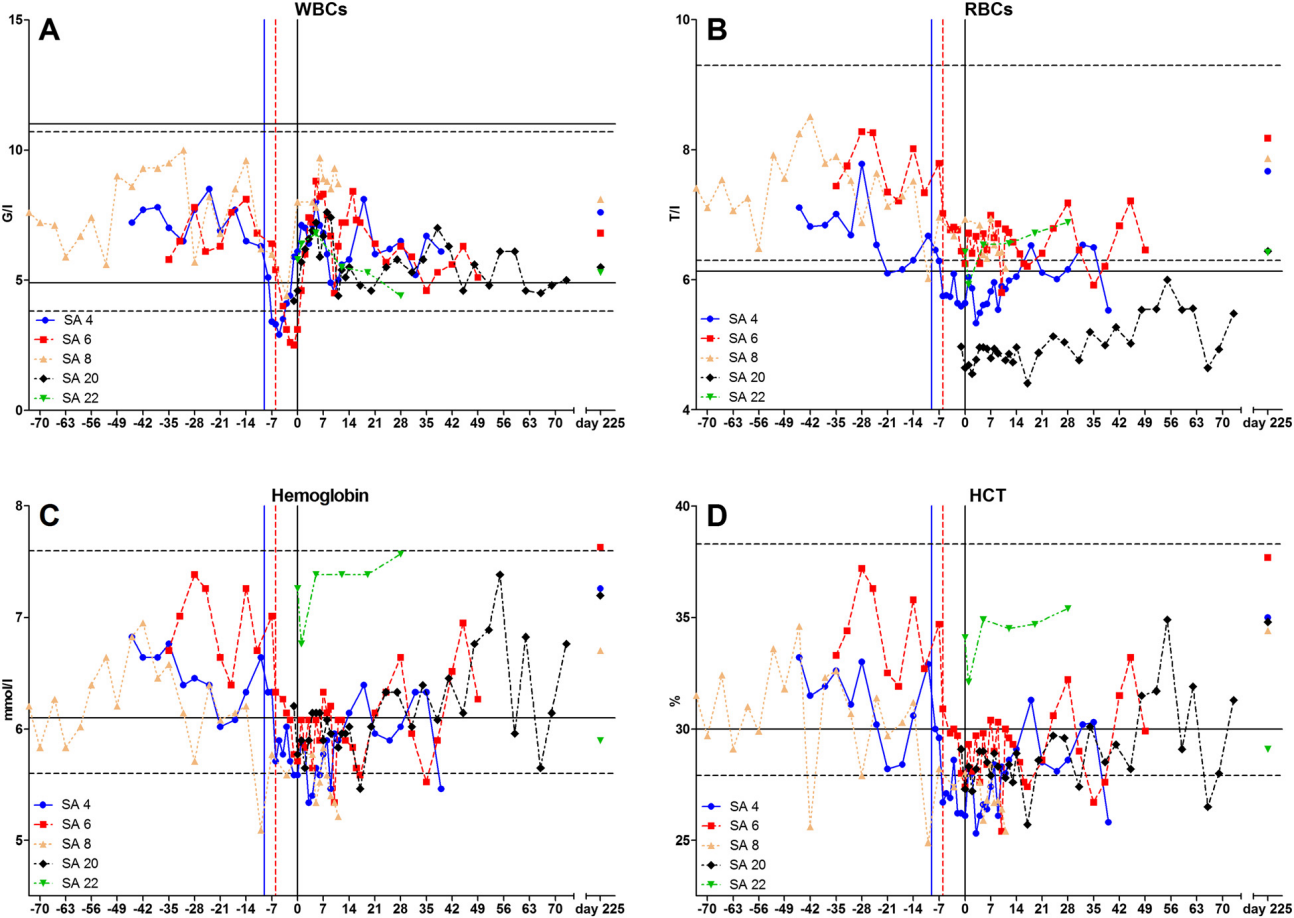
**Chronology of hematologic parameters of study animal (SA) 4, SA 6, SA 8, SA 20, and SA 22. A** Concentration of white blood cells (WBCs). **B** Concentration of red blood cells (RBCs). **C** Hemoglobin concentration. **D** Hematocrit (HCT). Blue continuous vertical line: Beginning of acute stage of SA 4. Red hashed vertical line: Beginning of acute stage of SA 6. Black continuous vertical line: Day of seroconversion. Hashed horizontal lines delimit Simmental reference range. Continuous horizontal lines delimit Limousin reference range (B-D only lower limit displayed).

Shortly before seroconversion, infected animals SA 4, SA 6, and SA 8 showed a period of decline in white blood cell (WBC) concentration followed by an increase. SA 20 and SA 22 showed only a consistent increase in WBC concentrations after seroconversion. In SA 4 and SA 6, the decline was below the Simmental reference range (Table 2) for four days shortly after entering the acute stage (Figure 1A). Reduced WBC concentrations consisted of an equal reduction in the concentrations of neutrophils and lymphocytes.

**Table 2:** **Mean, SD and percentiles for laboratory parameters of** ***B*** **.** ***besnoiti*** **negative Simmental cattle (9 animals, 224 samples)**

**Hematology**	**Mean ± SD**	**Percentiles**	
		**2.5th**	**97.5th**
RBCs (T/l)	7.45 ± 0.8	6.28	9.29
Hemoglobin (mmol/l)	6.5 ± 0.5	5.6	7.6
HCT (%)	32.6 ± 2.8	27.9	38.3
MCV (fl)	43.8 ± 3.1	36.1	48.9
MCH (fmol)	0.9 ± 0.1	0.7	1.0
MCHC (mmol/l)	20.0 ± 0.7	18.6	21.6
WBCs (G/l)	7.90 ± 1.7	3.80	10.70
**Serum chemistry**			
Total protein (g/l)	75.2 ± 4.7	66.3	83.0
Albumin (g/l)	34.8 ± 2.5	30.5	39.2
Globulin (g/l)	40.4 ± 5.2	29.2	49.3
A/G ratio	0.86 ± 0.16	0.64	1.24
Urea (mmol/l)	5.2 ± 0.9	3.5	6.8
Creatinine (μmol/l)	97.5 ± 16.5	70.4	128.2
Total bilirubin (μmol/l)	1.4 ± 0.7	0.1	2.8
Sodium (mmol/l)	142.3 ± 2.6	137.0	147.0
Potassium (mmol/l)	4.2 ± 0.4	3.4	5.0
Calcium (mmol/l)	2.4 ± 0.1	2.3	2.7
Phosphorus (mmol/l)	2.4 ± 0.3	1.8	3.1
Magnesium (mmol/l)	0.9 ± 0.1	0.8	1.1
Chloride (mmol/l)	100.8 ± 2.6	96.0	106.0
**Enzymes**			
AST (IU/l)	70.4 ± 9.7	50.6	89.4
GGT (IU/l)	18.9 ± 4.8	11.0	27.8
GLDH (IU/l)	12.5 ± 7.4	4.2	31.5
CK (IU/l)	217.0 ± 65.0	133.2	377.0

SD = standard deviation.

A decline in red blood cell (RBC) concentration was observed in SA 4 lasting for 20 days (Figure 1B). RBC concentrations of SA 20 fluctuated below the lower limit of the Limousin reference range (Table 3) for the whole trial period.

**Table 3:** **Mean, SD and percentiles of laboratory parameters of** ***B*** **.** ***besnoiti*** **seronegative and** ***B*** **.** ***besnoiti*** **seropositive Limousin cattle**

	**Seronegative**			**Seropositive**		
**Hematology**	**Mean ± SD**	**Percentiles**		**Mean ± SD**	**Percentiles**	
		**2.5th**	**97.5th**		**2.5th**	**97.5th**
RBCs (T/l)	8.44 ± 1.9^a^	6.13	13.70	**7.08 ± 1.2** ^**a,b**^	5.08	9.76
Hemoglobin (mmol/l)	7.5 ± 0.7	6.1	8.9	7.9 ± 1.3^a^	5.8	10.7
HCT (%)	35.5 ± 3.5	30.0	41.3	36.3 ± 5.6^a^	23.0	45.4
MCV (fl)	43.9 ± 9.4	25.9	58.1	**52.2 ± 3.9** ^**b**^	43.9	58.8
MCH (fmol)	0.9 ± 0.2	0.5	1.3	**1.1 ± 0.1** ^**b**^	0.9	1.3
MCHC (mmol/l)	21.2 ± 0.9	19.8	23.2	21.5 ± 1.4^a^	19.2	24.6
WBCs (G/l)	7.34 ± 1.6	4.90	11.00	7.31 ± 2.94^a^	2.83	14.44
**Serum chemistry**						
Total protein (g/l)	68.4 ± 5.9	60.1	81.7	**76.0 ± 6.9** ^**b**^	63.1	89.1
Albumin (g/l)	32.9 ± 4.4	26.0	41.2	**36.4 ± 2.7** ^**b**^	31.5	41.3
Globulin (g/l)	35.3 ± 4.3	28.7	43.8	**40.3 ± 7.0** ^**a,b**^	28.5	55.5
A/G ratio	0.95 ± 0.18^a^	0.73	1.39	0.94 ± 0.19^a^	0.63	1.38
Urea (mmol/l)	3.9 ± 1.3^a^	2.6	7.0	**5.2 ± 1.9** ^**a,b**^	2.2	9.6
Creatinine (μmol/l)	163.6 ± 21.2	124.7	207.7	164.4 ± 27.1^a^	125.4	214.6
Total bilirubin (μmol/l)	2.6 ± 0.9	1.0	4.4	2.9 ± 1.2^a^	0.9	5.5
Sodium (mmol/l)	145.8 ± 3.4	139.0	153.0	**141.1 ± 3.1** ^**b**^	135.5	146.5
Potassium (mmol/l)	5.3 ± 0.9	3.6	6.7	**4.8 ± 0.7** ^**b**^	3.4	6.0
Calcium (mmol/l)	2.3 ± 0.1	2.1	2.6	2.3 ± 0.2	1.9	2.6
Phosphorus (mmol/l)	1.6 ± 0.3	1.1	2.3	**1.9 ± 0.4** ^**b**^	1.3	2.8
Magnesium (mmol/l)	0.6 ± 0.2	0.3	1.1	**0.9 ± 0.1** ^**a,b**^	0.7	1.1
Chloride (mmol/l)	99.2 ± 2.3	95.6	103.2	**96.7 ± 2.6** ^**b**^	91.6	101.5
**Enzymes**						
AST (IU/l)	155.1 ± 61.9^a^	68.9	323.4	**102.2 ± 31.6** ^**a,b**^	58.2	166.0
GGT (IU/l)	16.6 ± 6.6	6.8	28.7	19.5 ± 9.1	1.7	35.4
GLDH (IU/l)	14.7 ± 7.1^a^	5.2	29.6	12.7 ± 8.1^a^	4.0	33.2
CK (IU/l)	702.8 ± 466.8^a^	78.0	2092.0	**331.9 ± 283.1** ^**a,b**^	106.5	885.0

Samples from *B. besnoiti* seronegative Limousins comprise 41 samples from 41 animals, samples from *B. besnoiti* seropositive Limousins comprise 75 samples (hematology) respectively 65 samples (serum chemistry/enzymes) from 54 animals. SD = standard deviation, ^a^ = normality test not passed, ^b^ = significantly (P < 0.01) different versus seronegative Limousins (Mann–Whitney-Test) highlighted in bold.

SA 4 and SA 6 showed a decline in hemoglobin concentration and HCT shortly before seroconversion. SA 20 showed strong fluctuation below or around the lower limit of the reference range for the whole trial period (Figure 1C and D).

Mean corpuscular hemoglobin concentration (MCHC) showed strong fluctuations above the lower limit of reference range in all infected animals (data not shown). Mean corpuscular volume (MCV) and mean corpuscular hemoglobin (MCH) concentrations were within the reference range in all infected animals with few borderline values (data not shown).

### Chronology of changes in serum chemistry during acute, subacute, and chronic bovine besnoitiosis

**Figure 2:**
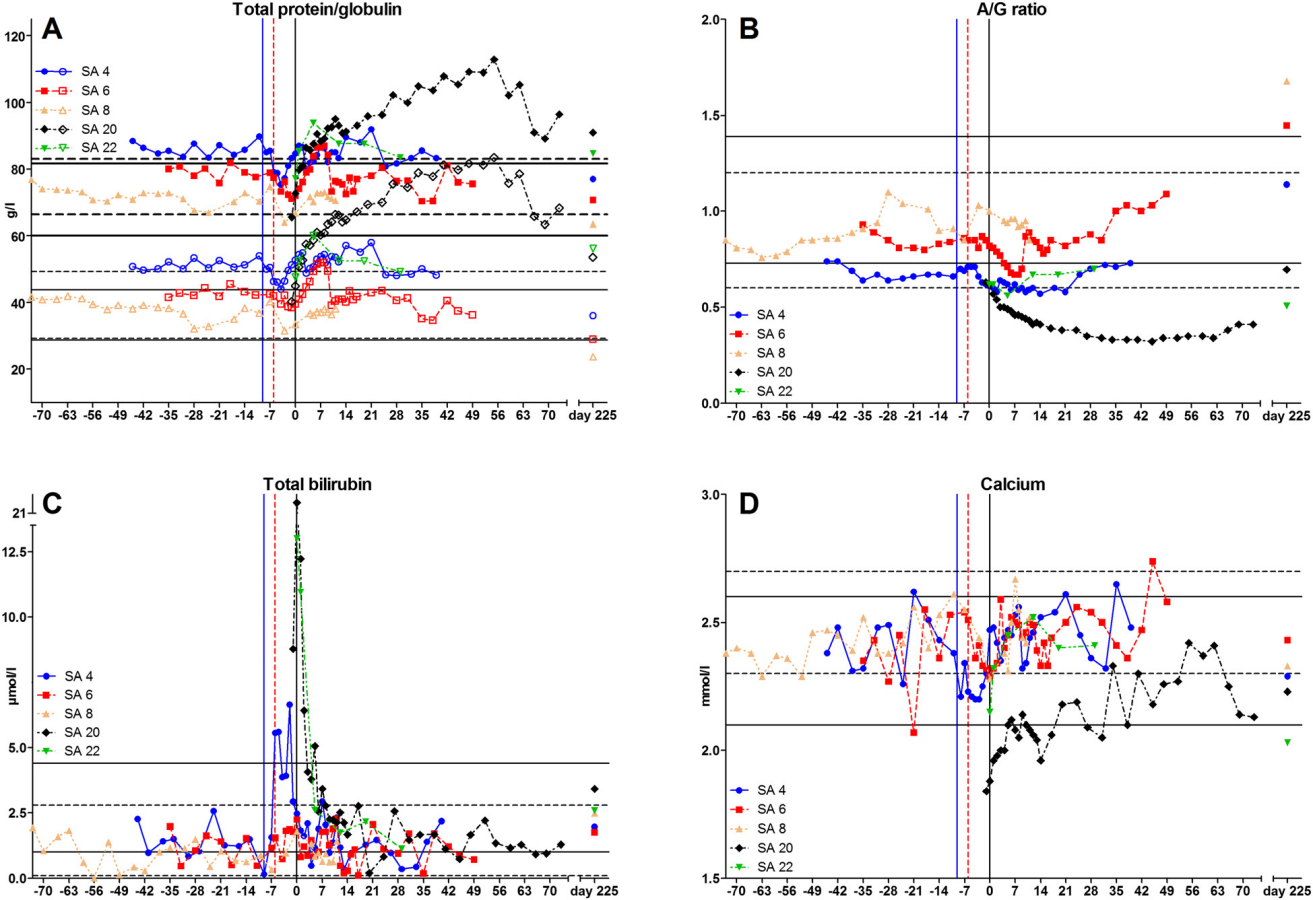
**Chronology of serum chemistry parameters of study animal (SA) 4, SA 6, SA 8, SA 20, and SA 22. A** Total protein (filled symbols) and globulin (empty symbols) concentrations. **B** Albumin/globulin (A/G) ratio. **C** Total bilirubin concentration. **D** Calcium concentration. Blue continuous vertical line: Beginning of acute stage of SA 4. Red hashed vertical line: Beginning of acute stage of SA 6. Black continuous vertical line: Day of seroconversion. Hashed horizontal lines delimit Simmental reference range (thick upper lines for total protein and thin lower lines for globulin). Continuous horizontal lines delimit Limousin reference range (thick upper lines for total protein and thin lower lines for globulin).

Elevations of TP and globulin concentrations plus decline in A/G ratio were detectable shortly after seroconversion in SA 4, SA 6, SA 20, and SA 22 (Figure 2A and B).

Albumin concentration declined within reference range shortly after the beginning of the acute stage in SA 4. SA 20 displayed fluctuating albumin concentrations around the lower limit of the reference range during the whole trial period. SA 20 and SA 22 showed increasing albumin concentrations for few days after seroconversion.

On day 225, albumin concentrations were within reference range in SA 20 and SA 22 and borderline in SA 4, SA 6, and SA 8.

Total bilirubin concentrations were above the reference range in SA 4, SA 20, and SA 22 (Figure 2C). In SA 20 and SA 22, this elevation was mainly caused by an elevated unconjugated bilirubin concentration (data not shown).

Urea concentrations were below the reference range for five days in SA 4 starting 4 days *ante* seroconversion (*das*) and for four days in SA 6 starting 4 *dps*. Lowest values were 2.8 mmol/l for SA 4 and 3.1 mmol/l for SA 6. SA 20 displayed an increased urea concentration on 1 *das* (7.9 mmol/l). SA 22 showed decreased urea concentration (2.4 mmol/l) for one day 5 *dps* (data not shown).

Creatinine concentrations were above the reference range in SA 4 for five days starting 8 *das* (147 μmol/l) and for three days starting 3 *das* in SA 6 (135 μmol/l). On day 225, total bilirubin, urea and creatinine concentrations were within the respective reference ranges in all animals except creatinine concentration in SA 22 (100.8 μmol/l) (data not shown).

Concentrations of magnesium (except SA 4, see below), phosphate, potassium, sodium and chloride displayed strong fluctuations within the respective reference ranges in all infected animals, with single values lying outside the upper or lower limits (data not shown). SA 4 displayed a decline of magnesium concentrations starting 6 *das* for four days below reference range. Calcium concentrations declined in all three Simmentals shortly before seroconversion and were below the reference range in SA 4 and SA 20 (Figure 2D).

### Chronology of serum protein gel electrophoresis during acute, subacute, and chronic bovine besnoitiosis

**Figure 3:**
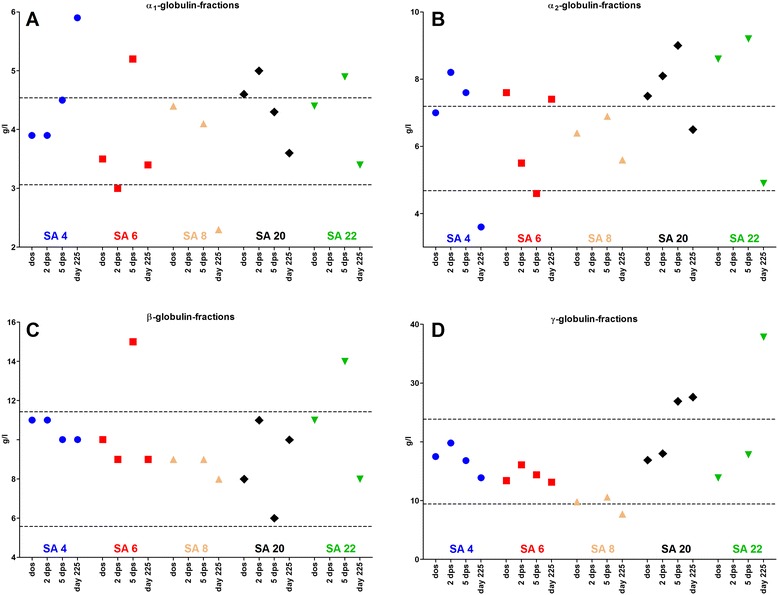
**Chronology of absolute concentrations of serum globulins of study animal (SA) 4, SA 6, SA 8, SA 20, and SA 22. A** α_1_-globulins. **B** α_2_-globulins. **C** β-globulins. **D** γ-globulins. Serum protein gel electrophoresis, horizontal lines delimit Simmental reference range.

During the acute stage, SA 4 displayed no alterations of serum protein fractions compared with the Simmental reference range (Table 4). SA 6 displayed a slightly elevated α_1_-globulin-fraction (7.1 g/l) and a diminished α_2_-globulin-fraction (2.4 g/l) 7 *das*. Alterations of serum protein fractions on the day of seroconversion and thereafter are depicted in Figure 3A-D.

**Table 4:** **Mean, SD and percentiles of serum gel electrophoresis of** ***B*** **.** ***besnoiti*** negative Simmental cattle (12 animals, 24 samples) and comparison with results from the *2-year-group (24 animals) of Alberghina et al. [25]

**Data obtained in the present study**				Literature data*
**Serum Gel Electrophoresis**	**Mean ± SD**	**Percentiles**		**Mean ± SD**
		**2.5th**	**97.5th**	
Total protein (g/l)	64.92 ± 3.55	59.58	70.85	68.10 ± 10.13
Albumin (%)	49.4 ± 4.6	40.3	56.1	Not given
α1-globulins (%)	5.7 ± 0.8	4.5	7.3	Not given
α2-globulins (%)	9.4 ± 1.4	6.5	11.2	Not given
β-globulins (%)	13.2 ± 2.1	8.2	16.7	Not given
γ-globulins (%)	22.4 ± 6.0	14.3	36.1	Not given
Albumin (g/l)	32.04 ± 3.35	25.45	36.85	31.79 ± 5.12
α1-globulins (g/l)	3.67 ± 0.42	3.06	4.54	5.92 ± 2.54
α2-globulins (g/l)	5.96 ± 0.68	4.68	7.19	5.77 ± 1.73
β-globulins (g/l)	8.54 ± 1.47	5.58	11.43	7.50 ± 1.16
γ-globulins (g/l)	14.55 ± 4.22	9.45	23.85	16.81 ± 3.72

SD = standard deviation.

### Chronology of enzyme activities during acute, subacute, and chronic bovine besnoitiosis

**Figure 4:**
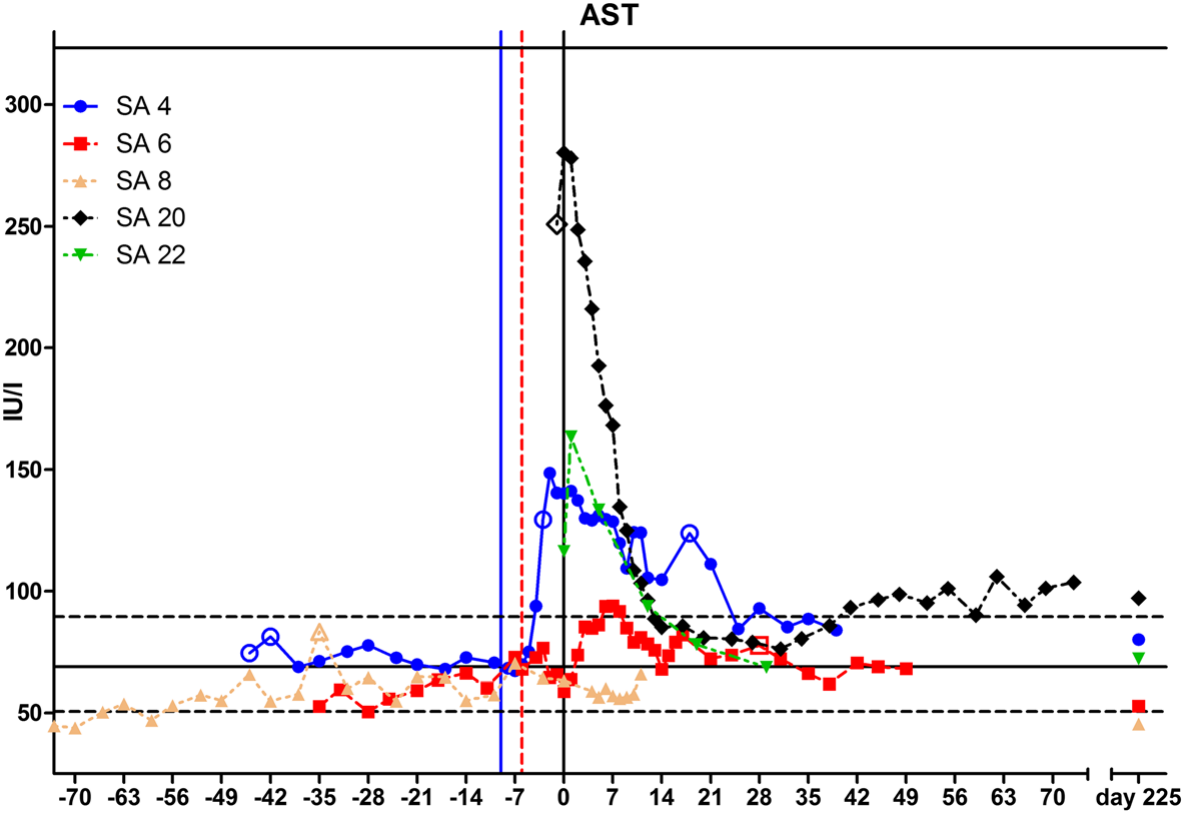
**Chronology of aspartate transaminase (AST) activities of study animal (SA) 4, SA 6, SA 8, SA 20, and SA 22.** The empty symbols depict the days on which creatine kinase activities were above the respective reference range. Blue continuous vertical line: Beginning of acute stage of SA 4. Red hashed vertical line: Beginning of acute stage of SA 6. Black continuous vertical line: Day of seroconversion. Hashed horizontal lines delimit Simmental reference range. Continuous horizontal lines delimit Limousin reference range.

AST activities are depicted in Figure 4.

CK activities were elevated for one day 3 *dps* in SA 4 (1,115 U/l) and 1 *das* in SA 20 (2,900 U/l). Serum activities of GGT and GLDH remained below the upper limit of the reference range in all infected animals.

On day 225, activities of GGT, GLDH, and CK remained below the upper limit of the reference range in all animals.

### Herd-BbGER1 monitoring: significant differences in laboratory parameters between *B. besnoiti* seronegative and *B. besnoiti* seropositive Limousin cattle

Means, standard deviations and percentiles of hematology, serum chemistry and enzyme activities for *B. besnoiti* seronegative and *B. besnoiti* seropositive Limousin cattle are shown in Table 3.

The RBC concentration was significantly higher and MCV and MCH were significantly lower in *B. besnoiti* seronegative Limousins. TP, albumin and globulin concentrations were significantly higher in *B. besnoiti* seropositive Limousins.

AST and CK activities were significantly lower in *B. besnoiti* seronegative Limousins (Table 3).

There were no statistical significant differences between parameters of *B. besnoiti* seropositive subclinically affected and *B. besnoiti* seropositive clinically affected Limousin cattle.

During chronic besnoitiosis, laboratory parameters of SA 20 and SA 22 were within the percentiles of the *B. besnoiti* seropositive Limousin group, except RBC and albumin concentrations which were lower in SA 20 and globulin concentration which was higher in SA 20.

## Discussion

The results of this study present the chronology of laboratory parameters during a longitudinal clinical trial monitoring five cattle with naturally acquired acute, subacute, and chronic bovine besnoitiosis. In addition, the laboratory parameters of *B. besnoiti* seronegative and *B. besnoiti* seropositive chronically infected Limousin cattle from a herd naturally affected by bovine besnoitiosis are compared.

Variations exist between reference ranges of different laboratories, cattle breeds, age groups, lactation and pregnancy status [26-31]. Thus, reference ranges for the evaluation of laboratory parameters of *B. besnoiti* infected Simmental cattle were calculated from values of healthy *B. besnoiti* negative Simmental study animals. Although larger animal cohorts are usually used for calculation, these reference ranges were regarded representative for this study, because housing, feeding and handling were similar and examination of cattle samples of the same breed were examined in the same laboratories. For the same reason, laboratory parameters of SA 20 and SA 22 were compared with reference ranges calculated from healthy *B. besnoiti* seronegative female Limousin cattle of Herd-BbGER1. Furthermore, we could not find appropriate up-to-date reference values for adult Limousin cows in the literature and application of older data could have caused misinterpretations because reference values may change due to genetic and environmental factors [32].

### The effect of bovine besnoitiosis on hematological parameters

Several hematological parameters were altered during acute bovine besnoitiosis. In the acute stage, leukopenia was observed in SA 4, SA 6, and SA 20. Leukopenia is a common finding in cattle with viral, bacterial and protozoan infections [33,34] and is due to the low storage neutrophil pool of adult cattle as well as increased margination and tissue emigration during acute inflammation.

For the low RBC concentrations in SA 4 and SA 20 there are two possible explanations: First, multifocal hemorrhages, which have been described in acute besnoitiosis [15] and were also clinically observed in SA 4 [23] and in multiple consecutive histological skin sections taken during acute besnoitiosis in these animals [35]. However, total bilirubin concentrations - although of limited value as a confirmation of hemorrhages in sick cattle (see below) - returned to the normal range after a few days, indicating that hemorrhages are not the only reason contributing to this finding. Second, anemia can also be a result of a chronic inflammatory disease [36,37]. The constantly low RBC concentrations of SA 20 and the lower RBC concentrations in the seropositive Limousin group are most likely caused by the chronic inflammatory state. Reticulocyte stains of blood smears to assess the origin of anemia were not performed and reticulocytes were not observed in conventionally stained smears. Similar significant differences in hematological parameters, namely increased MCV and MCH and decreased RBC concentrations were also observed in Iranian goats infected by *B. caprae* [21].

### The effect of bovine besnoitiosis on serum chemistry values

The initial hypothesis of increased globulin concentrations during chronic bovine besnoitiosis proved true. However, the effect of besnoitiosis on albumin concentrations was different than expected.

A decrease in albumin synthesis, albumin loss or hemodilution can be the cause of SA 20′s hypoalbuminemia [38]. However, clinical examinations and the results of laboratory tests did not reveal evidence for albumin loss or hemodilution. Two effects probably caused the decreased albumin synthesis in SA 20: inflammation and a negative energy balance. Albumin is a negative acute phase protein and inflammatory hypoalbuminemia can develop after on-going inflammatory states and is usually expected to be mild [38]. Moreover, it is tempting to speculate that the massive development of numerous *B. besnoiti* cysts in the tissues of SA 20 led to an increased demand in nutrients and subsequently to a negative energy balance. The hypocalcemia in this animal is very likely associated with hypoalbuminemia [39], as clinical signs of hypocalcemia were not observed.

The increased globulin concentrations are most likely due to increased antibodies against *B. besnoiti*, since reciprocal IFAT titers displayed a similar increase post seroconversion [23]. This hypothesis is supported by the electrophoresis findings displaying increased γ-globulin fractions 5 *dps* in SA 20 and on day 225 in SA 20 and SA 22.

The increase in the α_1_-fraction (α_1_-lipoprotein, α_1_-antitrypsin, and α_1_-antichymotrypsin) during the febrile phase of SA 6 and the increase in both α_1_- and α_2_-fractions (α_2_-macroglobulin and haptoglobins) of SA 4, SA 6, SA 20, and SA 22 shortly after seroconversion are most likely a response to the acute inflammation. Interestingly, the α-fraction was only elevated during the febrile phase in SA 6 on one day. A reason for this may be the rather mild clinical course of the disease in SA 4 and SA 6. The increase in the β-fraction of SA 6 and SA 22 on 5 *dps* is most likely due to an increase in IgM-antibodies or complement proteins, because transferrin, a main part of the β-fraction is expected to be low during acute inflammation [40]. SA 20′s elevated γ-globulin fraction (mainly IgG) displayed a two-peak pattern 5 *dps*, and elevated γ-globulin fractions of the other days displayed only one peak. This is indicative of a polyclonal hypergammaglobulinemia shortly after seroconversion changing into a monoclonal or oligoclonal gammoglobulinemia in the chronic stage.

The significant hyperproteinemia of *B. besnoiti* seropositive Limousin cows of Herd-BbGER1 compared with *B. besnoiti* seronegative Limousin cows is mainly caused by hyperglobulinemia. These findings correlate with the results obtained from the trial animals. Elevation of γ-globulin concentration is common during chronic inflammatory diseases [38], and similar results have also been reported in goats suffering from natural chronic caprine besnoitiosis [20]. Unexpectedly, albumin concentrations were higher in *B. besnoiti* seropositive Limousins. Albumin tends to decrease during inflammatory states (see above), and decreased albumin concentrations have also been found in cases of caprine besnoitiosis [41]. Maybe the higher albumin concentrations are due to a slight dehydration of these animals, which is also indicated by slightly higher urea concentrations of *B. besnoiti* seropositive Limousins compared with *B. besnoiti* seronegative Limousins. Severely infected Limousins in this study had a tendency to have higher total protein and globulin concentrations as well as lower A/G ratios. The former two observations were also made in goats naturally infected with *B. caprae* in Iran [20].

The hyperbilirubinemia of SA 4, SA 20, and SA 22 most likely has two main causes: A prehepatic icterus following hemorrhages (see above), or anorexia and sickness, which are common reasons for hyperbilirubinemia in different species (including cattle) and can include elevated unconjugated bilirubin concentrations [42-45]. The decrease in urea concentrations in SA 4 and SA 6, as well as low and borderline magnesium concentrations observed for 7 *das* in SA 4 can be associated with a period of anorexia in these animals, too. The elevation of creatinine concentrations in SA 4 and SA 6 are most likely due to dehydration [46]. SA 4 and SA 6 showed clinical dehydration on several days and the dehydration period coincides with the anorectic period [23]. Urea/creatinine ratio to further assess azotemia was not helpful in these cases, because urea concentrations were reduced.

Electrolyte concentrations in blood are influenced by feed intake, hydration status or sweating and are subject to daily change, as it was observed in the trial animals. These factors are also a plausible explanation for the occasional changes observed in the two Limousin groups. Using our data to conclude that bovine besnoitiosis affects electrolyte concentrations seems inappropriate at this point and further studies should be conducted to assess the importance of these alterations.

### The effect of bovine besnoitiosis on enzyme activities

The increased AST activities of SA 4 and SA 20 are most likely associated with a loss of muscle fiber integrity, because CK activities were elevated at the same time and GLDH and GGT displayed no elevated activities. The longer half-life of AST [47,48] explains the relatively rapid decline of CK activities and the slow removal of AST from serum. Muscle fiber damage may be caused by prolonged recumbence, because *B. besnoiti* affected cattle show apathy and are more likely to lie down and rest [49]. Or elevated AST activities could be a result of mild muscular degeneration and necrosis, lesions which are described in cattle during acute besnoitiosis after experimental infection with high parasite doses [15].

Although lower AST and CK activities are of little clinical significance, it is surprising that AST and CK activities were lower in *B. besnoiti* seropositive Limousins compared with *B. besnoiti* seronegative Limousins. Tissue cysts surrounded by severe chronic inflammatory reaction can be found in the musculature of diseased cattle [9], and loss of muscle fiber integrity due to cysts or inflammation seems likely. Maybe the effect of tissue cysts on muscle fiber integrity is too low to get noticed or leads to mild, gradual release of muscle enzymes, which cannot be detected due to the short half-life of CK. In comparison with the results obtained from the trial animals, loss of muscle fiber integrity seems to play only a role during acute and subacute besnoitiosis.

## Conclusions

This study provides detailed results of laboratory parameters obtained during the course of naturally acquired acute, subacute and chronic bovine besnoitiosis for the first time. Even though a low number of animals were examined in acute and subacute besnoitiosis, it could be shown that hematologic parameters were altered especially during acute and subacute besnoitiosis, leading to reduced red and white blood cell concentrations. Furthermore, during acute and subacute besnoitiosis, elevation of serum globulin fractions reflected the acute inflammatory state, clinical parameters like disturbed condition resulted in increased bilirubin concentrations and lesions like muscle necroses, which are described in the literature led to increased aspartate transaminase and creatine kinase activities. Chronic besnoitiosis led to reduced concentrations of red blood cells and to hyper-(gamma)-globulinemia most likely due to the chronic inflammatory condition caused by the parasite.

## Abbreviations

A/G ratio: Albumin/globulin ratio
AST: Aspartate transaminase
CK: Creatine kinase
das: Days ante seroconversion
dps: Days post seroconversion
GGT: Gamma-glutamyl transpeptidase
GLDH: Glutamate dehydrogenase
HCT: Hematocrit
Herd-BbGER1: First German cattle herd diagnosed with cases of bovine besnoitiosis
IFAT: Immunofluorescent antibody test
MCH: Mean corpuscular hemoglobin
MCHC: Mean corpuscular hemoglobin concentration
MCV: Mean corpuscular volume
RBCs: Red blood cells
SA: Study animal
td: Trial day
WBCs: White blood cells
